# Protein recycling pathways in neurodegenerative diseases

**DOI:** 10.1186/alzrt243

**Published:** 2014-03-06

**Authors:** Faisal Fecto, Y Taylan Esengul, Teepu Siddique

**Affiliations:** 1 Division of Neuromuscular Medicine, Davee Department of Neurology and Clinical Neurosciences, Northwestern University Feinberg School of Medicine, Tarry Building, Room 13-715, 303 East Chicago Avenue, Chicago, IL 60611, USA; 2 Interdepartmental Neuroscience Program, Northwestern University, Chicago, IL 60611, USA; 3 Department of Cell and Molecular Biology, Northwestern University Feinberg School of Medicine, Chicago, IL 60611, USA

## Abstract

Many progressive neurodegenerative diseases, including Alzheimer disease, Parkinson disease, Huntington disease, amyotrophic lateral sclerosis, and frontotemporal lobe dementia, are associated with the formation of insoluble intracellular proteinaceous inclusions. It is therefore imperative to understand the factors that regulate normal, as well as abnormal, protein recycling in neurons. Dysfunction of the ubiquitin-proteasome or autophagy pathways might contribute to the pathology of various neurodegenerative diseases. Induction of these pathways may offer a rational therapeutic strategy for a number of these diseases.

## Introduction

A common theme in neurodegeneration is the age-related accumulation of specific toxic protein species secondary to mutations or misfolding or both. Such disease-related proteins are prone to aggregation, and their oligomers cause toxicity via a gain-of-function mechanism [1]. The removal of misfolded or damaged proteins is critical for optimal cell functioning, particularly in neuronal cells, where the dynamic control of protein stability is crucial for synaptic development, function, and maintenance [2-6]. This dependence of neurons on protein recycling systems makes them particularly vulnerable to damage when these systems break down [7,8].

## Protein quality control in neurons

**Figure 1:**
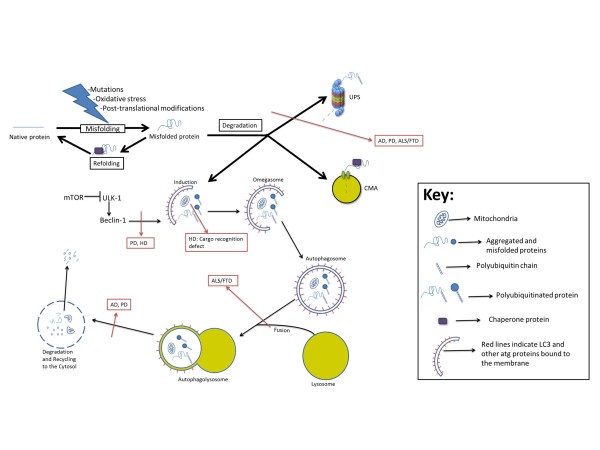
**Defects in protein recycling pathways in various neurodegenerative diseases.** For autophagy, the defect can be at the level of autophagy induction, cargo recognition, autophagosome/lysosome fusion, or lysosomal degradation. AD, Alzheimer disease; ALS, amyotrophic lateral sclerosis; CMA, chaperone-mediated autophagy; FTD, frontotemporal lobe dementia; HD, Huntington disease; mTOR, mammalian target of rapamycin; PD, Parkinson disease; ULK-1, Unc-51 like autophagy activating kinase 1; UPS, ubiquitin-proteasome system.

Neurons adapt to cellular stresses by maintaining the integrity of their proteome (Figure 1). This maintenance is regulated in three correlated ways by molecular chaperones: (a) refolding, (b) degrading, or (c) sequestering misfolded proteins into insoluble aggregates [1,9]. It is not yet understood how chaperones prioritize between these fates for cellular proteins. Chaperones are also essential in assisting newly synthesized proteins to fold properly and translocate across cellular membranes. When mechanisms regulating protein recycling go awry, as happens during aging, proteins begin to lose their conformation and their state becomes energetically more favorable for the formation of insoluble aggregates [1,10,11]. This is a common mechanism of pathogenesis in protein conformational diseases, including neurodegenerative diseases [12-14].

Long-term cellular stress can cause high levels of protein accumulation over time and this can lead to chaperone depletion and compromise the refolding of these accumulated proteins. When refolding is not sufficient to maintain cellular homeostasis in these conditions, misfolded proteins are transferred to the degradation pathways [15,16]. These proteins are handled by two systems: the ubiquitin-proteasome system (UPS) and autophagy. Even though the molecular machinery employed by each of these systems is unique, they essentially function in a similar manner to handle protein recycling. Each pathway has steps involved in cargo selection and tagging of the cargos, recognition and delivery of these cargos to the proteolytic machinery, degradation in the proteolytic core, and eventually recycling of the constituent amino acids [17].

The UPS is the primary intracellular proteolytic system responsible for the maintenance of rapid protein turnover in the cytosol and nuclei of cells, including the selective removal of abnormal and misfolded proteins. This degradation occurs in a two-step process: (a) the targeting of proteins through the covalent linkage of specific branched polyubiquitin chains and (b) the degradation of the tagged proteins by the downstream 26S proteasome complex. The 26S proteasome is composed of two sub-complexes: the 19S regulatory particle and the 20S core particle. In neuronal cells, dynamic control of protein stability is crucial for synaptic development and function, and the UPS plays an important role in the regulation of synaptic proteins [2]. It has been shown that depletion of the 26S proteasomes in mice leads to neurodegeneration; thus, they are essential for normal neuronal homeostasis and survival [18]. Aggregation-prone proteins may themselves impair the UPS by (a) saturating the capacity of the intracellular UPS machinery, (b) sequestering of essential components of the UPS into inclusions, or (c) clogging up the proteasome. Since the UPS is essential for cellular protein recycling, repeated attempts to degrade protein aggregates may hinder its ability to fulfill other physiologic tasks and this may ultimately lead to cellular dysfunction or death.

Autophagy represents an alternate pathway to the UPS for degradation of cytosolic proteins and organelles, including lipid structures and glycoproteins. Autophagy has a role in many biological processes, including nutrient recycling for prolonged survival during starvation and cytoprotection through degradation of insoluble inclusions in various neurodegenerative disorders. Since neurons are post-mitotic cells with polarized morphology and active protein trafficking, they are highly dependent on autophagy for their survival [3,4]. Furthermore, suppression of basal autophagy by knocking out autophagy-related genes leads to severe neurodegeneration in mice secondary to an accumulation of ubiquitin-positive protein aggregates and neuronal loss in the cerebellar cortex, hippocampus, and cerebellum [19,20]. Interestingly, impairment of the UPS can cause a compensatory increase in autophagy in order to maintain cellular homeostasis [21]. However, autophagy inhibition, in addition to affecting long-lived proteins, compromises the UPS function [22].

Autophagy can be subdivided into the macroautophagy-lysosomal system (MALS), chaperone-mediated autophagy (CMA), and mitophagy. MALS is responsible for bulk lysosomal degradation of cytosolic proteins, including protein aggregates and organelles. Mitophagy is the selective degradation of mitochondria. Unlike other types of autophagy, which rely on vesicle-mediated delivery of substrates to the lysosome, CMA is a selective process that involves direct translocation of substrates across the lysosomal membrane. When MALS is induced, a double-membrane compartment called an autophagosome sequesters an area of the cytoplasm and eventually fuses with the lysosome, where lysosomal enzymes degrade its contents. The MALS pathway has been implicated in several neurodegenerative diseases. As neurological disease progresses, there is an accumulation of autophagosomes or autophagic vacuoles in the diseased brains. In general, neurodegeneration is associated with defects at various steps in the autophagic process: (a) the disruption of autophagosome formation, (b) the disruption of autophagosome maturation, and (c) the disruption of autophagosome clearance.

Several adaptor proteins or shuttling factors are involved in the transport of protein substrates. Such proteins usually possess ubiquitin-associated (UBA) and ubiquitin-like (UBL) domains and include the ubiquilins (UBQLN) and SQSTM1/p62 [23]. They bind the substrates with their UBA domain and transport them to the proteasome, where they subsequently bind to the 19S regulatory particle with their UBL domain [23]. Interestingly, the UBQLNs and SQSTM1/p62 have also been proposed to form the link between UPS and autophagy [23].

We will now discuss the involvement of protein recycling pathways in the most common age-related neurodegenerative diseases, focusing on the UPS, MALS, and mitophagy pathways (Figure 1). CMA has been reviewed in detail by others and will not be the focus of this review [24].

## Alzheimer disease

Alzheimer disease (AD) is the most common cause of senile dementia and is characterized by progressive dementia accompanied by personality changes, psychosis, and language problems. Neuropathology is characterized mainly by extracellular senile plaques, consisting primarily of β-amyloid (Aβ), and intracellular neurofibrillary tangles, with hyperphosphorylated microtubule associated protein tau as a main constituent. Dystrophic neurites surrounding the plaques are ubiquitinated and consist of several ubiquitin-binding proteins, such as UBQLNs and SQSTM1/p62. There is not much evidence that the UPS is involved in degradation of Aβ or its precursor, the β-amyloid precursor protein (βAPP). On the other hand, tau is inefficiently degraded by the UPS, although the non-canonical 20S proteasomal degradation rather than the ubiquitin-dependent 26S proteasomal degradation seems to have a predominant role [25]. Despite the limited role for the UPS in the recycling of Aβ and tau, there is evidence that both of these proteins can impair UPS function [26,27] and such impairments have been observed in several brain regions of patients with AD [28]. Interestingly, a frame-shift mutant of ubiquitin B (UBB^+1^) has been found to accumulate in the dystrophic neurites and neurofibrillary tangles of AD [29] as well as other tauopathies and in polyglutamine diseases but not in synucleinopathies [30]. Although low levels of UBB^+1^ are degraded by the UPS, high levels of UBB^+1^ are incompletely degraded by the UPS, resulting in inhibition [31]. It has been reported that ubiquitination of UBB^+1^ is mediated by E2-25 K, which is essential for Aβ toxicity in animal AD models [32]. There is also emerging evidence that the ubiquitin-like protein, UBQLN1, is important in the regulation of βAPP and the AD-related protein, presenilin 1 [33].

Abundant autophagic vacuoles were first identified by electron microscopy studies on AD brain [34] and later confirmed by immunolabeling studies to be a major reservoir of intracellular Aβ [35]. This accumulation of autophagic vacuoles seems to be secondary to a combination of increased autophagy induction and their defective clearance [36,37]. Hyperactivation of mammalian target of rapamycin (mTOR) by Aβ results in decreased autophagy and contributes to tau hyperphosphorylation [38-40]. Furthermore, genetic inhibition of autophagy enhances Aβ-induced toxicity in cultured neurons [41]. Lee and colleagues [42] have previously reported that loss of presenilin 1 activity led to impairments in autophagosomal function as a consequence of lysosomal alkalinization, caused by failed maturation of the proton translocating V0a1 subunit of the vacuolar (H^+^)-ATPase and targeting to the lysosome. Several groups have supported these findings under presenilin 1 loss-of-function conditions [43-46]. However, these findings were not supported by two recent independent studies [47,48]. Reliable lysosomal pH measurement requires an appropriate probe and optimal experimental conditions and controls, and the differing observations by these groups may be due to differences in experimental approaches employed [44]. Interestingly, genetic enhancement of lysosomal activity has been shown to reduce Aβ accumulation and prevented the development of cognitive deficits in a mouse model of AD [49].

There is some evidence supporting a potential beneficial effect of inducing autophagy with rapamycin in AD [50]. For instance, it has been shown that rapamycin-mediated mTOR inhibition reduces Aβ and tau accumulation in an autophagy-dependent manner and reduces cognitive deficits in two different mouse models of AD [38,51]. Furthermore, induction of autophagy by viral vector-mediated expression of beclin1, which is involved in autophagic vesicle nucleation, reduces Aβ pathology in a mouse model of AD [52]. However, it must be noted that a possible dysregulation of mTOR-protein synthesis by rapamycin may have detrimental effects on learning and memory [53,54]. Also, if autophagosome clearance is impaired, as has been suggested by a lysosomal acidification defect in AD, autophagy induction may cause further accumulation of autophagosomes and autophagy substrates. For instance, it has been shown that rapamycin treatment decreases lifespan of flies overexpressing Aβ42 [55]. Collectively, these observations suggest that UPS and autophagy play a critical role in the pathogenesis of AD and that mTOR or beclin 1 signaling or the activation (or a combination of these) may be important therapeutic targets in AD.

## Parkinson disease

Parkinson disease (PD) is a progressive neurodegenerative disorder caused by a selective death of dopaminergic neurons in the substantia nigra and results in a resting tremor, rigidity, slowness of movement, and postural instability. The characteristic pathology of PD includes cytoplasmic inclusions called Lewy bodies, whose major constituent is α-synuclein. It has been reported that the UPS is the main degradation pathway for α-synuclein under normal conditions *in vivo* while, with increased α-synuclein burden, MALS is recruited [56]. It has been shown that expression of the A53T mutant of α-synuclein induces alterations of the UPS [57]. Providing further evidence for a role of the UPS in PD pathogenesis, Bedford and colleagues [18] reported that depletion of the 26S proteasome leads to an accumulation of α-synuclein and the development of Lewy-like inclusions. There is also limited evidence for a direct genetic role for UPS involvement in PD pathogenesis. A putative causal mutation in ubiquitin C-terminal hydrolase L1 has been identified in two German siblings with PD [58]. Mutations in the E3 ligase parkin cause juvenile PD [59,60], and the mechanism was linked to diminished affinity for the 19S particle for at least some of the mutations [61]. Parkin is reportedly involved in proteasomal degradation of several substrates, including α-synuclein, and in mitophagy.

Autophagy has a vital role to play in the pathogenesis of PD. Conditional deletion of the autophagy-related protein, Atg7, in the substantia nigra dopaminergic neurons recapitulates many of the pathologic features of PD [62]. Moreover, it has been shown that α-synuclein inclusions are preferred targets for SQSTM1/p62-dependent autophagy [63]. The familial PD-linked proteins, such as α-synuclein, DJ-1, parkin, PINK1, and LRRK2, are also known to be involved in the autophagic pathway. For instance, it is known that overexpression of wild-type α-synuclein impairs macroautophagy by causing mislocalization of the autophagy protein, Atg9, and decreased formation of autophagosome precursors called omegasomes [64]. In addition, overexpression of the A53T mutant of human α-synuclein induces an accumulation of autophagic vacuoles [57,65]. Functional deficiency of DJ-1 also leads to altered autophagy via an increase in autophagic flux in murine and human cell lines [66]. Moreover, cells transfected with mutant LRRK2 show marked accumulation of autophagic vacuoles [67]. The full-length PINK1 interacts with beclin 1, and it has been shown that the overexpression of PINK1 significantly enhances both basal and starvation-induced autophagy. Of note, the W437X mutant of PINK1 shows an impaired interaction with beclin 1 and lacks the ability to induce autophagy [68]. Interestingly, it has been shown that beclin 1 gene transfer activates autophagy and ameliorates the neurodegenerative pathology in an α-synuclein model of PD [69].

It is known that damaged mitochondria accumulate with normal aging. Mitochondrial dysfunction has also been implicated in the pathogenesis of PD. It has been shown that parkin is selectively recruited to damaged mitochondria and is important in their selective elimination [70]. Moreover, damage to mitochondria facilitates the rapid accumulation of PINK1, a mitochondrial serine/threonine kinase, in the mitochondria which recruits parkin to induce mitophagy in a process which involves the recruitment of SQSTM1/p62 and VDAC1 [71,72]. This PINK1/parkin-mediated mitophagy is compromised by PD-linked mutations [73]. Consistent with this, it has been shown that there is an accumulation of damaged mitochondria in knockout models of PINK1 and parkin [74-76].

Mutations in genes encoding lysosomal proteins, such as glucocerebrosidase (GBA) and lysosomal type 5 P-type ATPase (ATP13A2), have also been linked to PD [77]. GBA mutations impair lysosomal function, leading to α-synuclein accumulation, which further decreases lysosomal GBA activity by impairing the trafficking of GBA from the endoplasmic reticulum-Golgi to lysosomes, leading to neurodegeneration [77]. Recent work has shown that deficiency of ATP13A2 leads to lysosomal dysfunction, α-synuclein accumulation, and neurotoxicity [78,79]. Collectively, these observations suggest that impairments of UPS, autophagy, and mitophagy may underlie PD pathogenesis and that these pathways may serve as relevant targets for the design of rational therapies in PD.

## Amyotrophic lateral sclerosis and frontotemporal lobe dementia

Amyotrophic lateral sclerosis (ALS) is the most common form of motor neuron disease and is caused by selective degeneration of motor neurons in the brain and spinal cord. Progressive weakness leads to paralysis that is ultimately fatal, in most cases, within 5 years of symptom onset. About 5 % of patients with ALS develop features of overt and disabling dementia, usually of the frontotemporal lobar type (FTD) [80]. The presence of ubiquitin-positive proteinaceous inclusions in motor neurons is the signature pathological feature of ALS, and it has been proposed that dysfunction of the UPS might play a role in this phenomenon. Indeed, motor neuron-specific disruption of proteasomes results in an ALS-like phenotype in mice [81]. Functional alterations of the UPS occur in motor neurons of mutant SOD1-linked ALS mice and may play a role in disease progression [82]. It is also known that mutant SOD1 is degraded by the proteasome and that partial inhibition of proteasome activity leads to the formation of large SOD1-containing aggregates [83-87].

Direct etiological evidence for the involvement of UPS and MALS pathways was provided by the identification of mutations in UBQLN2 and SQSTM1/p62 in ALS and FTD [23,88,89]. Pathologic inclusions, containing ubiquitin, SQSTM1/p62, and UBQLN2, are a common feature in a wide spectrum of ALS and ALS-FTD, implying a functional convergence at the level of abnormal turnover of ubiquitinated proteins [23,80,88]. Indeed, it has been shown that mutant UBQLN2 impairs UPS function *in vitro*[88], implying that induction of UPS function can be a viable therapeutic option in ALS and ALS-FTD.

Although a defective UPS has been suggested to produce ALS-associated protein aggregates, recent studies have revealed a prominent role for autophagy [90]. Surviving motor neurons from mutant SOD1-linked ALS mice at the end stage of disease show accumulation of autophagic vacuoles within the cytosol in a progressive and protein aggregation-related manner [91]. Mutant SOD1 can be recognized by SQSTM1/p62 in a ubiquitin-independent fashion and targeted for autophagy [92]. Autophagy induction clears aggregated proteins and rescues motor neuron degeneration in ALS mice [90,93]. However, it has also been shown that rapamycin treatment in mutant SOD1-linked ALS mice resulted in exacerbation of ALS pathology, earlier disease onset, and faster disease progression [94]. Moreover, although lithium-mediated autophagy induction was originally reported to be beneficial in both mice and patients with ALS [91], these findings could not be reproduced by subsequent studies [95-97]. Interestingly, a recent study has suggested a potential use of trehalose and enhancers of mTOR-independent autophagy for the treatment of ALS [98]. Therefore, further studies are required to clarify the potential role of autophagy induction in ALS.

An autophagy defect has also been suggested by genetic studies of ALS and FTD. For instance, mutations in UBQLN2 and SQSTM1/p62 have been reported in ALS and FTD [88,89,99]. UBQLNs were previously thought to be involved in protein degradation via the UPS. Recently, several studies have provided convincing evidence for their role in autophagy [100-102]. UBQLNs are present in autophagosomes and bind LC3 in a complex [101,103]. Overexpression of UBQLNs protects cells from starvation-induced death (via autophagy and UBA-domain dependent mechanisms), whereas depletion renders cells more susceptible [100]. Depletion of UBQLNs also regulates formation and maturation of autophagic vacuoles [100,101]. It has been reported that SQSTM1/p62 binds ubiquitinated proteins and LC3 [104]. Under conditions of impaired autophagy, SQSTM1/p62 mediates the aggregation of ubiquitinated proteins and sequesters them from the UPS [22,105]. Similarly, mutations in VCP and OPTN cause ALS [106,107]. VCP mutations also cause the syndrome of inclusion body myopathy with Paget disease of bone and FTD [108]. Interestingly, both VCP and OPTN are also involved in protein degradation via autophagy [109-111]. Recently, the ALS-linked protein FIG 4 was also shown to be involved in autophagy [112]. Similarly, mutations in the endosomal ESCRTIII-complex subunit CHMP2B cause FTD [113]. VCP and ESCRT family members, including CHMP2B, are known to participate in autophagosome-lysosome fusion.

The ALS-FTD-associated protein TDP43 is degraded by both the UPS and autophagy pathways, and it has been shown that overexpression of SQSTM1/p62 reduces TDP43 aggregation in an autophagy- and proteasome-dependent manner [114-116]. Deficiency of VCP and ESCRT family members has been shown to lead to the accumulation of the TDP43 [109,117,118]. Interestingly, overexpression of UBQLN1 has been shown to potentiate the aggregation of TDP43 and modify toxicity in a drosophila model of ALS [119,120]. Future studies to address the precise molecular mechanism of UPS and autophagy dysfunction in ALS and FTD are essential to identify appropriate therapeutic targets.

## Huntington disease

Huntington disease (HD) is caused by a polyglutamine repeat expansion in the huntingtin (htt) gene, resulting in protein aggregation and causing a syndrome of involuntary movements and dementia. Although it is clear that the proteasome is involved in the degradation of mutant htt, the role of proteasomes remains contradictory [121]. Interestingly, a recent unbiased screen has identified both UBQLN1 and UBQLN2 to be highly associated with htt inclusions [122]. Altered autophagy has been observed in post-mortem specimens from HD patients and animal models [123,124]. It has been suggested that wild-type htt may function physiologically as an autophagy regulator, and it has been shown that cells expressing mutant htt have an increase in autophagic vacuoles [125]. A cargo recognition defect has been suggested as the mechanism underlying autophagic dysfunction in HD [126]. Another mechanism may be related to recruitment of beclin 1 by mutant htt, which impairs beclin 1-mediated long-lived protein turnover [127]. The involvement of autophagy in HD has been further demonstrated by the sequestration of mTOR in polyglutamine aggregates in cell models, transgenic mice, and human brains. This sequestration of mTOR impairs its kinase activity and induces autophagy. This mechanism protects against polyglutamine toxicity, as autophagy induction attenuates htt accumulation and cell death in cell models of HD, whereas the inhibition of autophagy has opposite effects [128-130]. Thus, the therapeutic induction and recovery of autophagy may be useful to enhance the clearance of mutant htt and reduce its neurotoxicity.

## Conclusions

Because of their post-mitotic nature, polarized morphology and complex arborization, neurons are particularly sensitive to alterations in protein homeostasis. A plethora of evidence has firmly established the essential role for the UPS and autophagy pathways in both normal physiological functioning as well as pathophysiological conditions of the nervous system.

Impairments in the quality control of proteins and organelles in the neuronal soma, axon, and synapses led to an aggregation of toxic proteins and, thereby, cause neuronal dysfunction and neurodegeneration in diseases such as AD, PD, ALS, FTD, and HD.

As reviewed above, several independent studies have shown that autophagy induction can be beneficial as a disease-modifying treatment in experimental models of various neurodegenerative diseases. Of note, rapamycin has been in clinical practice for several years. But as a disease-modifying therapy in neurodegeneration, long-term use of rapamycin may have adverse effects on cognition by dysregulating physiological mTOR signaling, which is important for axonal growth, dendritic arborization, and synaptic plasticity [50,131]. However, such adverse effects have not been reported so far in patients undergoing long-term rapamycin therapy. Autophagy induction therapy would have to be initiated either before disease onset or in the very early stages before pathological protein aggregates have become too large to overwhelm the cellular protein recycling pathways. Moreover, understanding the step(s) affected in the autophagic process is essential to developing targeted therapeutic approaches based on the modulation of autophagy. For instance, if autophagosome-lysosome fusion or lysosomal acidification is impaired, autophagy induction would lead to an accumulation of non-degradable autophagosomes and this could be detrimental. It should also be noted that long-term induction of autophagy may reduce cell viability by reducing the cells’ responsiveness to stress [50].

Mechanistic correlates for abnormal UPS and autophagy in these diseases have yet to be fully understood. Since the cargo destined for recycling may need to be transported over great distances in a neuron, it is likely that complex processes involving abnormal protein-protein interactions or impaired trafficking (or both) may be involved. A better understanding of these mechanisms will aid in the design of rational therapies for neurodegenerative diseases.

## Abbreviations

AD: Alzheimer disease; ALS: amyotrophic lateral sclerosis; ATP13A2: lysosomal type 5 P-type ATPase; Aβ: β-amyloid; CMA: chaperone-mediated autophagy; FTD: frontotemporal lobar dementia; GBA: glucocerebrosidase; HD: Huntington disease; htt: huntingtin; MALS: macroautophagy-lysosomal system; mTOR: mammalian target of rapamycin; PD: Parkinson disease; UBA: ubiquitin-associated; UBB+1: frame-shift mutant of ubiquitin B; UBL: ubiquitin-like; UBQLN: ubiquilins; UPS: ubiquitin-proteasome system; βAPP: β-amyloid precursor protein.

## Competing interests

The authors declare that they have no competing interests.
